# Chromatin State-Based Analysis of Epigenetic H3K4me3 Marks of *Arabidopsis* in Response to Dark Stress

**DOI:** 10.3389/fgene.2019.00306

**Published:** 2019-04-03

**Authors:** Hengyu Yan, Yue Liu, Kang Zhang, James Song, Wenying Xu, Zhen Su

**Affiliations:** ^1^State Key Laboratory of Plant Physiology and Biochemistry, College of Biological Sciences, China Agricultural University, Beijing, China; ^2^College of Life Sciences, Qingdao University, Qingdao, China; ^3^Key Laboratory of Hebei Province for Plant Physiology and Molecular Pathology, College of Life Sciences, Hebei Agricultural University, Baoding, China; ^4^Henan Experimental High School, Zhengzhou, China

**Keywords:** H3K4me3, extended darkness, chromatin state, SOM analysis, *Arabidopsis*

## Abstract

Light is essential to plant growth and development. Extended darkness causes dramatic gene expression changes, leading to leaf senescence, hypocotyl growth, petiole elongation, reduced leaf area, and early flowering, etc. However, the underlying mechanism of response to darkness at epigenetic levels remains largely unknown. In this study, we conducted ChIP-seq to generate global epigenomic profiles of H3K4me3 under 3-day extended darkness and normal light conditions in *Arabidopsis*. We applied chromatin state analysis together with self-organization mapping (SOM) to study the combination of epigenetic regulation under dark stress. The SOM map clusters the segments on the genome according to multiple diverse epigenomic datasets, which breaks the limit of dispersed distribution of epigenetic marks on the genome. Through SOM analysis, we also found that the signals of H3K4me3 were mainly increased after darkness. Analysis of H3K4me3-changed genes together with differentially expressed genes indicated that the genes showing dark-increased H3K4me3 were most involved in senescence and autophagy, and cross-talk existed between dark-induced and natural senescence. In summary, we studied the regulation of the epigenetic H3K4me3 marks of *Arabidopsis* in response to dark stress using chromatin state and SOM analyses. Our study revealed the regulatory mechanisms of the epigenome in response to dark stress, and SOM analysis based on chromatin states used in our study will also be helpful for other studies on dynamic changes of multiple epigenetic marks.

## Introduction

Light is essential for plant growth and development. Thus, an increasing number of researchers have focused on the regulatory mechanism of light on plant growth and development. Mature plants need light for photosynthesis for energy production, which maintains normal growth and development of plants. Extended light deprivation induces significant phenotypic changes. For example, extended darkness triggers leaf senescence in *Arabidopsis* (Weaver et al., 1998; Weaver and Amasino, 2001; Lin and Wu, 2004). Shade light with low red/far red ratios triggers hypocotyl growth, petiole elongation, reduced leaf area, hyponastic leaf movement, fewer branches, leaf senescence, and early flowering (Casal, 1996; Cerdan and Chory, 2003; Finlayson et al., 2010; Kozuka et al., 2010; Casal, 2013).

Recently, the signaling pathways linking light deprivation and actual senescence processes were identified. Phytochrome-interacting factors PIF4 and PIF5, which are inhibited by phytochrome B (phyB) (Sakuraba et al., 2014), are reported to be required in the dark-induced leaf senescence in multiple coherent feed forward loops (Sakuraba et al., 2014; Zhang et al., 2015; Liebsch and Keech, 2016). PIF4 and PIF5 regulate ABA INSENSITIVE 5 (ABI5)/ENHANCED EM LEVEL (EEL) and ETHYLENE-INSENSITIVE3 (EIN3) through abscisic acid (ABA)- and ethylene-mediated signaling pathways, respectively. ABI5/EEL and EIN3 promote the expression of major senescence-associated genes, such as *ORESARA 1* (*ORE1*, also known as *ANAC092*), *NON-YELLOW COLORING1* (*NYC1*) and *STAY-GREEN* (*SGR*) to promote leaf senescence (Sakuraba et al., 2014; Qiu et al., 2015). PIF4 and PIF5 also directly bind to the G-box motifs located in promoters of senescence-associated genes, such as *ORE1*, *SGR* and *NYC1*, to regulate their expression (Sakuraba et al., 2014; Zhang et al., 2015). ORE1 also binds to *NYC1*, *NON-YELLOWING1* (*NYE1*), and *NON-YELLOWING2* (*NYE2*) promoters and stimulates their expression (Qiu et al., 2015). Another important dark-induced senescence regulator, *WRKY22*, may also be activated by PIFs (Zhou et al., 2011; Liebsch and Keech, 2016). In addition, the transcription factor bZIPs, such as bZIP63, which are involved in energy homeostasis, have functions in dark-induced leaf senescence resulting from activation of SNF1-RELATED PROTEIN KINASE 1 (SnRK1)-dependent signaling. *Arabidopsis* *atg* mutants, such as *atg5*, show an early leaf yellowing phenotype during dark-induced leaf senescence (Thompson et al., 2005), and autophagy-associated genes are also upregulated during dark-induced leaf senescence in addition to natural senescence (van der Graaff et al., 2006), indicating that autophagy is important for the remobilization and recycling of nutrients during leaf senescence (van der Graaff et al., 2006). In addition, our previous study showed that the transcriptional repressor JASMONATE-ZIM-DOMAIN PROTEIN 7 (JAZ7) was involved in dark-induced leaf senescence (Yu et al., 2015). The molecular regulatory mechanism in response to darkness has been largely reported, but the epigenetic regulation under dark stress is less studied.

Increasing evidence indicates that plant growth and development are governed by transcriptional and epigenetic regulation. The regulation of various genes can be affected by chromatin remodeling. Recently, an increasing number of researchers have demonstrated the regulation of epigenetic marks involved in leaf senescence. For example, overexpression of a chromatin-modifying AT-hook protein, *AT-HOOK MOTIF NUCLEAR-LOCALIZED PROTEIN 27* (*AHL27*), also known as *ORESARA 7* (*ORE7*), resulted in delayed senescence, indicating that chromatin modifications may be associated with changes in gene expression during leaf senescence (Lim et al., 2007). Overexpressing the histone methyltransferase *SU(VAR)3-9 HOMOLOG 2* (*SUVH2*) results in senescence delay, and histone 3 lysine 4 trimethylation (H3K4me3) is significantly increased around *WRKY53*, a key regulator during leaf senescence. Genome-wide analysis based on high-throughput sequencing is a powerful technique for identifying epigenetic characterization across the genome and is highly effective in making comparisons between different development stages and environmental conditions. For example, ChIP-seq (chromatin immunoprecipitation sequencing) has been widely used to explore histone modifications at the genome level (Barski et al., 2007; Ho et al., 2011; Du et al., 2013). Brusslan et al. (2012, 2015) successfully studied the genome-wide scale of H3K4me3 marks using ChIP-seq at different time points during age-induced developmental senescence to identify genes with increased H3K4me3 and upregulation in older leaves, indicating that H3K4me3 plays important roles in senescence. Hence, epigenetic marks may also play important roles in response to darkness, which triggers leaf senescence and other developmental processes.

Chromatin states, which represent a combination of epigenetic markers, impact the activity of gene expression during developmental processes and in response to environmental cues. Many studies have identified chromatin states to discover epigenetic regulatory mechanisms (Roudier et al., 2011; Luo et al., 2013; Sequeira-Mendes et al., 2014; Wang et al., 2015). The epigenetic marks were combined and dispersed in the genome. Hence, it is difficult to investigate the relationship between epigenetic marks and changes under different conditions. Self-organization mapping (SOM) map is used to effectively integrate, visualize, and mine diverse epigenomic data sets and provide a powerful way to identify complex relationships between epigenetic marks (Mortazavi et al., 2013). Recently, we integrated public and in-house epigenetic data sets in *Arabidopsis* to identify chromatin states and construct a new visualization tool, a SOM map in the Plant Chromatin State Database (PCSD) (Liu et al., 2018), which is powerful for studying the coordinated regulation of epigenetic marks and dynamic changes in response to different environmental conditions.

In previous study, we found that DNase I hypersensitive sites (DHSs) were diminished in euchromatin and increased in heterochromatin after extended darkness, which suggested that chromatin dynamics played an important role in response to darkness (Liu et al., 2017). We found that photosynthesis-associated genes were DHS-diminished and downregulated, but thousands of genes remained upregulated after extended darkness (Liu et al., 2017). In this study, we conducted a high-throughput sequencing technique for the epigenome to generate global H3K4me3 profiles and used chromatin state-based SOM analysis to study the dynamic changes of epigenetic H3K4me3 marks in *Arabidopsis* plants after extended dark treatment.

## Materials and Methods

### Plant Materials and Growth Conditions

*Arabidopsis thaliana* (Col-0) seeds were sown in soil. The seeds were stratified for 3 days at 4°C and then transferred to a conditioning chamber under 16 h light (22°C)/8 h dark (19°C) cycles. *Arabidopsis* plants were grown in soil for 1 month. All plants subjected to dark treatment were wrapped in aluminum foil. *Arabidopsis* plants were grown under normal light condition (see above) as a control. After 3 days, *Arabidopsis* shoots with and without extended dark treatment were harvested for ChIP-seq and RNA-seq.

### ChIP-Seq Analysis

ChIP-seq experiments were performed as previously described (Zhang et al., 2017) using anti-trimethyl-histone H3 (Lys 4) (H3K4me3, Millipore, 07-473). ChIP-seq libraries were developed from shoots under extended darkness and normal conditions and were sequenced by the Beijing Genomics Institute. Bowtie2 software (Langmead and Salzberg, 2012) was used to align the sequencing reads of ChIP-seq to the *Arabidopsis* reference genome (TAIR10) using default parameters. The peak in different conditions and differentially changed peaks were called by MACS software (Zhang et al., 2008). The nomodel parameter was set, and the *d*-value parameter was set at 200. The resulting wiggle files, which represent counts of ChIP-Seq reads across the reference genome, were normalized for sequencing depth by dividing the read counts in each bin by the millions of mapped reads in each sample and were visualized in the UCSC genome browser (Jiang et al., 2017; Weintraub et al., 2017; Zhang et al., 2017). The CEAS software (Shin et al., 2009) was used to analyze the distance between TSSs of genes and the nearest called peaks. H3K4me3 peaks located in the region 2 kb upstream of TSSs and gene bodies were considered H3K4me3-associated genes. The differential regions showing H3K4me3 modifications between dark stress and control conditions were also called by MACS software with the *d*-value parameter of 200. We then identified genes with differentially changed H3K4me3 peaks located in the region 2 kb upstream of TSSs and gene bodies and added identified genes from two replications together as H3K4me3-changed genes. Sequence data were deposited in the National Center for Biotechnology Information (NCBI) SRA database (accession number: PRJNA520815).

Quantitative real-time PCR analysis of ChIPed DNA (ChIP-qRCR) was performed on the Applied Biosystems 7500 Real Time PCR System. The gene-specific primers for other genes were shown in Supplementary Table 5. In order to determine the relative fold enrichment (RFE) of modified histone-associated sequences in the bound fractions, *ACT2* was used as positive control, as it shows a higher enrichment of H3K4me3, and 25S was used as negative control. RFE was calculated as 2^-ΔΔCT^ ± standard deviation (SD), where ΔΔCT = ΔCT (positive control) - ΔCT (negative control).

### SOM Analysis

The trained SOM maps used were from the Plant Chromatin State Database (PCSD) (Liu et al., 2018). The average signal values of epigenomic data in each segment were calculated using the bigWigAverageOverBed program of the UCSC Genome Browser (Kent et al., 2002). Then, the new data were mapped to the trained SOM map by a mapsom program in ERANGE software (Mortazavi et al., 2013). The comparison between the two SOM maps was performed by the diffmap program in ERANGE software.

### RNA-Seq Analysis

Total RNA was extracted using TRIzol reagent (Invitrogen) and purified using RNeasy Mini Kits (Qiagen). RNA samples were from *Arabidopsis* shoots under extended darkness treatment and normal light conditions. RNA-seq libraries were sequenced by the Beijing Genomics Institute. Sequencing reads of RNA-seq were aligned to the *Arabidopsis* genome (TAIR10) using TopHat software (Trapnell et al., 2009). Genes showing statistically significant differential expression on the basis of (log_2_ fold change > 1, *q*-value < 0.05) were identified as DEGs by using DESeq2.

### Functional Analysis

GO enrichment analysis was performed using the agriGO website (Du et al., 2010) and REVIGO (Supek et al., 2011). The gene set enrichment analysis was performed using PlantGSEA website (Yi et al., 2013).

### Phenotyping Experiments and Measurement of Chlorophyll Pigments

*Arabidopsis thaliana* (Col-0) seeds were surface-sterilized and sown on half-strength MS media supplemented with 1.5% sucrose or without sucrose. Ten-day-old plants were used for 7 days of extended darkness treatment (wrapped in aluminum foil) with normal light condition as a control. For measurement of total chlorophyll (Chl) content, Chl pigments were extracted with 80% acetone from leaf tissues of plants. The Chl concentration (mg/L) was determined using an ultraviolet/visible spectrophotometer according to the following formula:

Chlorophyll a: Ca = 12.21A_663_ – 2.81A_646_

Chlorophyll b: Cb = 20.13A_646_ – 5.03A_663_

Total chlorophyll: CT = Ca+ Cb

## Results

### Epigenetic Profiling of H3K4me3 Marks in Response to Darkness

To explore the regulatory mechanisms of epigenetic marks in response to darkness, in this study, we performed histone 3 lysine 4 trimethylation (H3K4me3) ChIP-seq using *Arabidopsis* whole plants exposed to 3-day darkness treatment and normal light conditions, with two independent biological replicates. *ACT2*, as the reference gene, was shown equal H3K4me3 enrichment signal in control and dark-treated tissue (Supplementary Figure 1). We identified a total of 14,827 and 13,921 enriched regions of H3K4me3 under dark stress and control conditions in biological replication 1, respectively and 13,758 and 13,469 enriched regions of H3K4me3 under dark stress and control conditions in biological replication 2, respectively (Supplementary Table 1). There were more enriched regions of H3K4me3 under dark stress than under control conditions.

**FIGURE 1:**
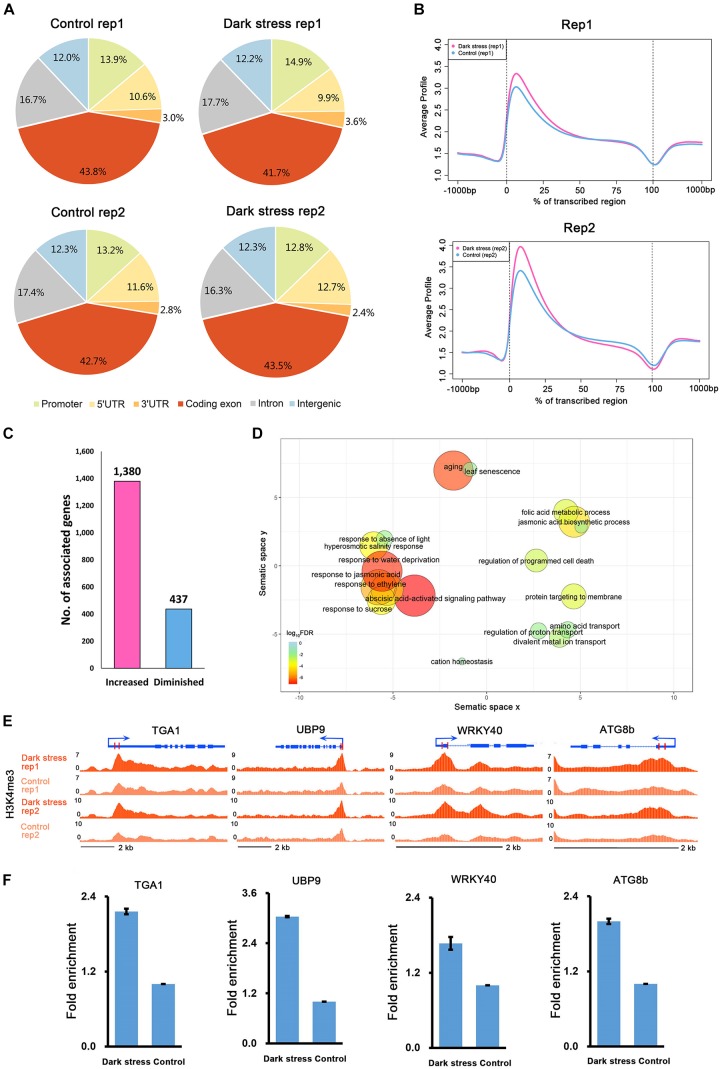
The characteristics of H3K4me3 in *Arabidopsis* under extended darkness and normal light conditions. **(A)** Genome-wide distribution of H3K4me3 within different regions under extended darkness and control conditions. **(B)** The profiles of H3K4me3 in *Arabidopsis* genes, including 1 kb upstream and downstream. Meta-gene profiles were generated using the normalized sequencing density of H3K4me3. The *X*-axis shows that the gene body was converted into a percentage to standardize genes of different lengths. Regions 1-kb upstream and downstream of the gene are included. The *Y*-axis represents average values of H3K4me3. **(C)** The number of genes with changed H3K4me3 signals in response to dark stress. **(D)** GO enrichment analysis of H3K4me3-increased genes by agriGO and REVIGO. The scatterplot shows the cluster representatives in a two dimensional space derived by applying multidimensional scaling to a matrix of significant GO terms with semantic similarities. Bubble color and size indicates the log_10_ (FDR *P*-value). **(E)** Locations of primers for H3K4me3 validation shown in UCSC genome browser. **(F)** ChIP–qPCR validation for H3K4me3.

To investigate the characteristics of H3K4me3, we first examined the distribution of H3K4me3 in the genome under each condition. We divided the *Arabidopsis* genome into six subclasses: promoter, 5′UTR, 3′UTR, coding exon, intron, and intergenic regions. The distributions of the H3K4me3 locations relative to *Arabidopsis* genes under the two conditions were similar (Figure 1A). Generally, there was a higher percentage of H3K4me3 in exon regions than in other genomic regions in these two different conditions. Meta-gene profiles of the H3K4me3 generated along the generic region showed that H3K4me3 was distributed downstream of transcription start sites (TSSs), which was similar under both conditions (Figure 1B), and the signal intensities of H3K4me3 were higher under dark stress than under control condition. Then, we identified genes with these differential H3K4me3 regions located within 2 kb upstream of TSS and gene bodies as H3K4me3-changed genes. We identified a total of 1,380 genes that had higher H3K4me3 signals after dark treatment than the control as H3K4me3-increased genes and 437 genes that had lower H3K4me3 signals after dark treatment than the control as H3K4me3-diminished genes in two biological replicates (Figure 1C and Supplementary Table 2). Average profiles for H3K4me3-changed genes showed that H3K4me3-increased genes had higher H3K4me3 signals and H3K4me3-diminished genes had lower H3K4me3 signals under dark stress than control conditions, which showed that our results were reliable (Supplementary Figure 2). The number of H3K4me3-increased genes was much greater than that of H3K4me3-diminshed genes. All of these results showed that the H3K4me3 was increased after extended dark treatment. Through GO enrichment analysis, it was found that the H3K4me3-increased genes were associated with aging, senescence, response to the absence of light and some processes closely related to hormones such as ABA, jasmonic acid (JA) and ethylene (Figure 1D and Supplementary Table 3), and the H3K4me3-diminished genes were associated with the response to gibberellin stimulus and response to UV light (Supplementary Table 4). To verify the ChIP-seq results, we selected some genes for ChIP-qPCR validation (Figure 1E,F) (Primers used shown in Supplementary Table 5). The ChIP-qRCR results of the selected genes confirmed the ChIP-seq results. *ATG8b*, *TGA1*, *UBP9* and *WRKY40* showed higher H3K4me3 after darkness than the control in the ChIP-seq results, and H3K4me3 increased after dark treatment in ChIP-qPCR results.

### SOM Analysis for Epigenetic Changes of H3K4me3 in Response to Darkness

Through the above analysis of the difference of H3K4me3 in response to darkness by MACS software, we found that H3K4me3 had an increasing trend under extended darkness compared to normal light condition. Next, we expected to confirm this change of H3K4me3 in response to darkness. Normally, the epigenetic marks located in the genome are dispersed, which makes it difficult to clearly display the relationship and changes of epigenetic marks on the genome, whereas the cluster of epigenetic marks on the genome is beneficial for investigating the changes of epigenetic marks. Hence, we needed visualization tools with clustering regions that contain epigenetic marks to display the genome-wide changes of multiple epigenetic marks and analyze their biological functions. Mortazavi’s group developed self-organizing maps (SOMs) to effectively integrate, visualize, and mine diverse genomics data types, including complex chromatin signatures (Mortazavi et al., 2013). They used 72 ChIP-seq and DNase-seq datasets to train the SOM map and analyzed transcriptional enhancers by comparing the chromatin signature from different cell types. The SOM maps trained by epigenomic data are a powerful way to identify complex relationships in genomic data. Recently, we integrated public and in-house epigenetic data sets for *Arabidopsis* to identify chromatin states, which represent multiple combinations of epigenetic marks, and constructed a new visualization tool, the SOM map in the Plant Chromatin State Database (PCSD) (Liu et al., 2018), which is powerful in the study of the dynamic changes of epigenetic marks in response to different environmental conditions (Mortazavi et al., 2013).

The SOM map in PCSD shows a reorganized genome. We integrated public epigenetic data sets, including DNase-seq, ATAC-seq, ChIP-seq and meDIP-seq, to define chromatin states based on the ChromHMM algorithm in *Arabidopsis thaliana.* Thirty-six chromatin states were defined, including 290,553 segments with combinational epigenetic marks across the whole genome in *Arabidopsis thaliana* (Supplementary Figure 3A). To cluster these segments with similar chromatin states, all of these segments with different chromatin states were mapped onto a 30 × 45 SOM map, which was trained with 100,000 iterations based on collected epigenetic data sets’ signals on each segment (Supplementary Figure 3B). In the trained SOM maps, the segments with similar chromatin states were clustered into the same or adjacent units. Therefore, the trained SOM map is a useful visualization tool to display a reorganized genome in which clustered genome segments contain a similar combination of epigenetic marks based on multiple epigenetic data sets. The custom epigenetic data can be mapped to a trained SOM map according to the signals of every segment to show the distribution of custom epigenetic mark signals on the SOM map. Due to the clustering segments being based on epigenetic signals, the major changes of one epigenetic mark under two different conditions can be displayed clearly by comparing two SOM maps of epigenetic marks under each condition.

**FIGURE 2:**
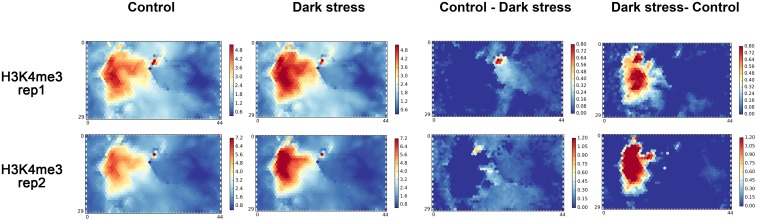
The SOM analysis for two biological replication datasets of H3K4me3 in response to extended darkness. The ChIP-seq data in different conditions are mapped to a trained SOM map in PCSD, which represents a re-organizing genome through clustering genome segments based on multiple epigenetic signals. “Control - Dark” represents the result of subtracting signals under the dark treatment from signals under control condition. “Dark - Control” represents the result of subtracting signals under the control condition from signals under the dark treatment.

To further explore the changes of H3K4me3 in response to darkness, we used a chromatin state-based SOM map with clustering regions that contain epigenetic marks to display the changes of H3K4me3 in response to darkness. We mapped our two biological replications to a trained SOM map based on the epigenetic signals for each segment, which resulted from the PCSD mentioned above. First, we investigated the location of units with high signals of H3K4me3 in the SOM map (Figure 2). H3K4me3 is located in the left-center. The distribution of H3K4me3 in treatment and control conditions is similar. The combined data of two repeats mapped to the trained SOM map showing similar results (Supplementary Figure 4A). Then, we explored the differential units of epigenetic H3K4me3 marks in the SOM map under dark stress and control conditions. We compared the SOM maps from the two conditions by subtracting scores in every unit of the SOM maps (Figure 2). Through SOM comparison between H3K4me3 under treatment and control conditions, we found that the units with major changes of H3K4me3 preferentially had higher scores under dark stress than under control conditions, and these units with major changes were located in H3K4me3’s characteristic locations. The SOM comparison for combined data of two repeats also showed increased H3K4me3 in characteristic locations (Supplementary Figure 4A). Therefore, the comparison of H3K4me3’s SOM maps between treatment and control conditions indicated that H3K4me3 modifications were increased at their characteristic locations under extended darkness compared to normal light conditions.

We also mapped our published data of DNase I hypersensitive sites (DHSs) in response to darkness onto the SOM map (Liu et al., 2017). DHSs are located in the top left corner and bottom left corner. DHSs had partial overlaps with H3K4me3 (Supplementary Figure 4B). These characteristic locations of H3K4me3 and DHSs were similar to other H3K4me3 and DHS locations in PCSD. By comparing the differential SOM maps in DHSs between treatment and control conditions, we found that the units with major changes located in DHSs’ characteristic location in SOM maps and these units had higher scores in control conditions than in dark stress (Supplementary Figure 4A). The results showed that DHSs were diminished under dark stress, which was consistent with our previous study (Supplementary Figure 4C).

### Senescence-and Autophagy-Associated Genes With Consistent Changes of H3K4me3 and Expression in Response to Darkness

**FIGURE 3:**
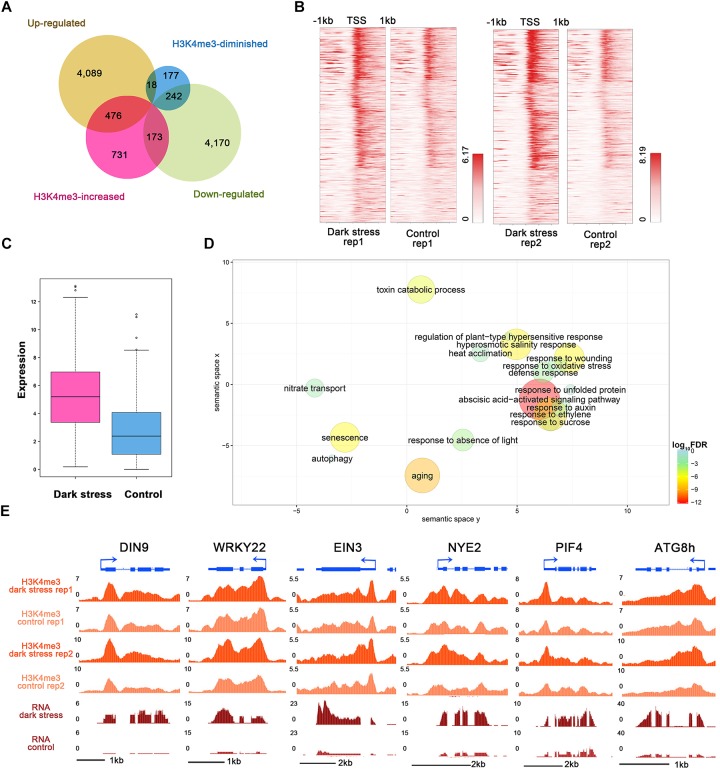
Integrated analysis of H3K4me3-changed and differentially expressed genes. **(A)** Venn diagrams between H3K4me3-changed genes and differentially expressed genes after dark treatment. **(B)** H3K4me3 signal values around TSSs of the group of 476 genes in **(A)** under dark treatment and control conditions, respectively. For each gene, the H3K4me3 signals are displayed along –1 kb to 1 kb regions around the TSSs. **(C)** The gene expression values are shown for the group of 476 genes in **(A)**. **(D)** GO enrichment analysis of 476 genes in **(A)** by agriGO and REVIGO. The scatterplot shows the cluster representatives in a two-dimensional space derived by applying multidimensional scaling to a matrix of significant GO terms with semantic similarities. Bubble color and size indicates the log_10_ (FDR *P*-value) (legend in bottom right-hand corner). **(E)** Genes involved in senescence and autophagy associated with H3K4me3 expression in the UCSC genome browser.

To study the influences of H3K4me3 histone modifications on expression, we conducted RNA-seq using the same materials as those used for the ChIP-seq experiment (Supplementary Table 6) and integrated ChIP-seq with RNA-seq datasets for plants in response to darkness. We identified 4,583 upregulated and 4,585 downregulated genes after 3 days of darkness (Supplementary Table 7). To explore the regulated genes by histone modification, we compared the groups of H3K4me3-changed genes and differentially expressed genes (Figure 3A). The majority of the changes in H3K4me3 signals were increased under dark stress; therefore, we focused on 476 H3K4m3-increased and up-regulated genes (Supplementary Table 8), which showed higher values of H3K4me3 signals (Figure 3B) and expression levels (Figure 3C) after dark treatment, thereby indicating a positive relationship between H3K4me3 and gene expression for these 476 genes. In addition, there were 173 genes with increased H3K4me3 signals and downregulated expression under dark stress. Due to the positive relationship between H3K4me3 and expression in the *Arabidopsis* genome, we mainly analyzed the potential function of these 476 H3K4me3-increased and upregulated genes under dark stress. Through GO enrichment analysis, we found that GO terms were associated with the response to the absence of light, aging and senescence (Figure 3D and Supplementary Table 9). Some processes closely related to hormones, which are closely related to senescence, such as ABA and ethylene, were also significantly enriched (Liebsch and Keech, 2016). Among these 476 genes with H3K4me3-increased and upregulated genes, 230 genes were reported as senescence-associated genes in the leaf senescence database (Supplementary Table 10) (Li et al., 2014). Among them, some genes had been confirmed to have functions in promoting senescence, such as *WRKY6*, *SAG113*, and *SAG101*; some genes were reported to be involved in dark-induced leaf senescence, such as *ABI5*, *EIN3* and *ORE1* (Liebsch and Keech, 2016). Thus, H3K4me3 plays an important role in the transcription activity of senescence-associated genes. The UCSC genome browser also showed that genes (*DIN9* and *WRKY22*) involved in leaf senescence had H3K4me3 gains and upregulated expression under dark stress downstream of TSSs (Figure 3E).

In addition, the GO term of autophagy was significantly enriched (Figure 3D and Supplementary Table 9). Many autophagy-associated genes had increased H3K4me3 under dark stress (Supplementary Table 11). For example, ATG8B and ATG8H (Figure 1E, 3E), which are involved in the ubiquitin-like ATG8 and PE conjugation pathways for autophagosome formation (Han et al., 2011), were H3K4me3-increased and upregulated after dark treatment. In addition, most genes involved in autophagy had upregulated expression under dark stress (Supplementary Table 11). Autophagy is involved in energy homeostasis under carbon starvation, as demonstrated by high-throughput metabolomic, lipidomic, and proteomic analyses (Avin-Wittenberg et al., 2015). Energy-associated GO terms, such as response to sucrose, were also enriched. These results showed that changes in H3K4me3 are accompanied by autophagy, which functions in energy homeostasis during dark-induced leaf senescence. In addition to GO enrichment analysis, we also found that these genes with increased H3K4me3 and upregulation had functions in autophagy and energy starvation by analyzing gene set enrichment by PlantGSEA (Yi et al., 2013). These 476 genes with increased H3K4me3 and upregulation in expression were enriched in the gene sets of increased-regulation by SNF1 KINASE HOMOLOG 10 (KIN10, also known as SNF1-RELATED PROTEIN KINASE 1.1, SNRK1.1), and the gene sets induced by KIN10 overlapped with those induced by starvation conditions and were antagonized by increased sugar availability (Supplementary Table 12). KIN10 plays an important role in linking stress, sugar and developmental signals to promote survival under darkness and sugar deprivation (Baena-Gonzalez et al., 2007). Eighty-eight KIN10-induced genes showed increased H3K4m3 and upregulation after dark treatment (Supplementary Table 12). These genes are involved in autophagy, development, response to stress, hormone metabolism, etc. Among these genes, dark-induced genes such as *DIN2*, *DIN6*, *DIN9* and *DIN10* induced by KIN10 all displayed more H3K4me3 deposition in addition to more expression in the dark treatment plants than in the control. These dark-induced genes also showed upregulation under other diverse stress conditions, such as sucrose starvation. A total of 73 of 88 genes with increased H3K4me3 showed the same expression changes as DINs that were upregulated under other various stresses, such as sugar starvation, in addition to darkness (Supplementary Table 13). These 73 genes were also repressed in expression when glucose or sucrose treatment was applied. These results indicated that changes in H3K4me3 occur in energy homeostasis during dark-induced leaf senescence.

To further study energy homeostasis during dark-induced leaf senescence, we performed an experiment to investigate *Arabidopsis* plants under different growth conditions as follows: plants were grown in 1/2 MS with normal sucrose or 1/2 MS with no sucrose, both under extended darkness and normal light conditions (Supplementary Figure 5). The leaves of *Arabidopsis* plants grown on plates without sucrose were smaller and contained less chlorophyll than those on plates with normal 1/2 MS. Under 7-day darkness, the plants subjected to both sucrose conditions had lower chlorophyll levels than plants under normal light condition, but the plates without sucrose reduced more chlorophyll than the plates with normal sucrose conditions, which indicated that lower energy supply and darkness accelerated leaf senescence.

Through the overlap of H3K4me3-changed and differentially expressed genes, we also found 242 genes with H3K4me3-diminished and downregulated genes, which also had a positive relationship between H3K4me3 and expression (Figure 3A and Supplementary Table 8). We also analyzed enriched GO terms in 242 H3K4me3-diminished and downregulated genes, which are involved in the glucosinolate biosynthetic process, the starch biosynthetic process, mRNA modification, the pigment biosynthetic process and response to UV light (Supplementary Table 14).

### Comparison of H3K4me3 Changes During Dark-Induced and Age-Triggered Leaf Senescence

**FIGURE 4:**
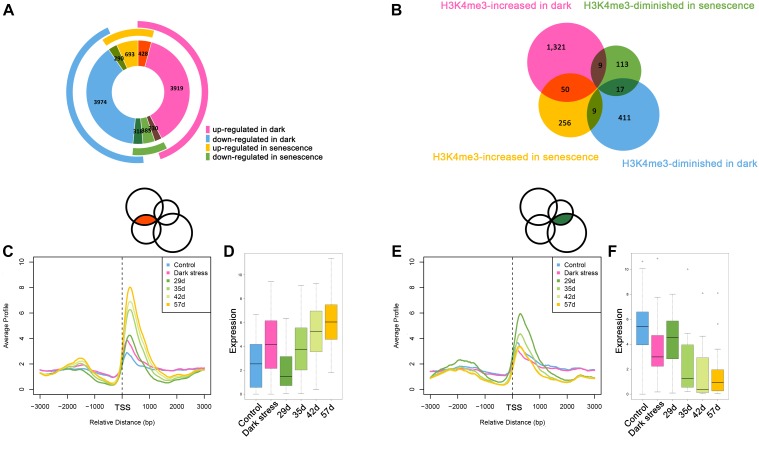
Comparative analysis between dark-induced leaf senescence and age-triggered leaf senescence. **(A)** The overlaps of differentially expressed genes during dark-induced leaf senescence and natural senescence. **(B)** The overlaps of H3K4me3-changed genes during dark-induced leaf senescence and natural senescence. **(C)** H3K4me3 signal values around TSSs of the 50 H3K4me3-increased genes during dark-induced and natural senescence from **(B)**. **(D)** Expression changes of the 50 H3K4me3-increased genes during dark-induced and natural senescence from **(B)**. **(E)** H3K4me3 signal values around TSSs of the 17 H3K4me3-diminished genes during dark-induced and natural senescence from **(B)**. **(F)** Expression changes of the 17 H3K4me3-diminished genes during dark-induced and natural senescence from **(B)**.

The above results showed that H3K4me3 played an important role in dark-induced leaf senescence. It was reported that there was cross-talk between dark-induced leaf senescence and age-triggered leaf senescence. To investigate the association between dark-induced leaf senescence and age-triggered natural senescence, we compared the genes involved in dark-induced leaf senescence and age-triggered leaf senescence (Figure 4A). The 428 genes that were upregulated after dark treatment also had higher expression in senescent samples than in younger samples. A total of 318 genes with downregulation after dark treatment also had lower expression in senescent samples than in younger samples. These results also showed cross-talk between dark-induced leaf senescence and age-triggered leaf senescence.

The changes in H3K4me3 have been reported to be associated with changes in gene expression during age-triggered leaf senescence (Brusslan et al., 2015), which attracted us to explore whether the H3K4me3-changed genes during dark-induced leaf senescence also had changes in H3K4me3 during natural senescence. Then, we compared the consistent and opposite changes in H3K4me3 during dark-induced leaf senescence and age-triggered leaf senescence. We still found some overlapping genes with changed H3K4me3 signals during dark-induced and age-triggered leaf senescence. More genes showed increased H3K4me3 both under darkness and natural senescence than those with opposite changes of H3K4me3 during these two senescence processes (Figure 4B). There were 50 H3K4m3-increased genes under dark stress and in senescent plants (Supplementary Table 15). In addition to higher values of H3K4me3 signals (Figure 4C), these genes also displayed higher FPKM values (Figure 4D) in both dark-treated and senescent plants, thereby indicating the same changes of H3K4me3 and gene expression between in darkness and natural senescence for the 50 genes. Among the 50 genes, many are involved in the salicylic acid (SA)/JA crosstalk, including *NAC019*, *WRKY75*, *GRXS13*, *GRX480*, *GLYI4*, and *RAP2.6* (Ndamukong et al., 2007; Krishnaswamy et al., 2011; Zheng et al., 2012; Guo et al., 2017; Proietti et al., 2018) (Supplementary Table 15). *GRXS13* and *GRX480* are involved in maintaining cell redox homeostasis, which could be inhibited by JA and are induced by SA (Ndamukong et al., 2007; La Camera et al., 2011). NAC019 directly suppresses the expression of *ICS1*, a key gene in SA biosynthesis (Zheng et al., 2012). NAC019 also regulates leaf senescence through the JA-mediated signaling pathway (Zhu et al., 2015). WRKY75 can positively regulate JA and SA-mediated plant immune responses (Chen et al., 2013; Choi et al., 2014), and can promote leaf senescence by enhancing SA content in *Arabidopsis* (Guo et al., 2017). These results suggest that the SA/JA signaling pathway mediated crosstalk between natural and dark-induced leaf senescence at the H3K4me3 level. In addition, there were 17 H3K4me3-diminished genes after darkness treatment and during natural senescence (Figure 4B and Supplementary Table 16), which had the same changes as H3K4me3 between dark-induced leaf senescence and age-triggered leaf senescence (Figure 4E) and displayed lower FPKM values both in darkness-treated and senescent plants (Figure 4F). In these 17 genes, there were genes associated with the auxin response, such as *SAUR64*, *SAUR63* and *SAUR20* (Supplementary Table 16). The crosstalk of H3K4me3 between dark-induced leaf senescence and age-triggered leaf senescence suggested that H3K4me3 was involved in leaf senescence in response to dark stress.

## Discussion

### SOM Analysis of the Changes of Epigenetic H3K4me3 Marks in Response to Dark Stress

In this study, we found that H3K4me3 was increased under extended darkness. To more directly investigate the dynamic change of H3K4me3, we used a new integrated visualization tool based on diverse epigenomic datasets, SOM. The SOM map clusters the segments on the genome according to multiple diverse epigenomic datasets, which breaks the limit of dispersed distribution of epigenetic marks on the genome. Hence, we mapped our epigenomic datasets, including ChIP-seq and DNase-seq data, in response to dark stress on trained SOM maps to clearly investigate the relationship of epigenetic marks and major changes under different conditions at the genome level. We found that different epigenetic marks had different major changes in response to dark stress by SOM analysis. The major changes of these epigenetic marks were located at their own characteristic locations. The SOM maps showed that H3K4me3 was mainly increased after darkness. We also integrated DNase-seq data in response to dark stress and found that DHSs were mainly diminished after darkness using SOM maps, which was consistent with our previous study. These results showed that chromatin state and SOM analyses are a powerful way to investigate the dynamic changes and coordinated regulation of multiple epigenetic marks of *Arabidopsis* in response to dark stress. Although SOM maps show the clustered segments with major changes of each epigenetic marker, the related genes in units of the SOM map contain some genes that may not have significant changes in epigenetic marks. Therefore, we need to calculate the significantly changed peaks by MACS software to identify related genes more strictly.

### The Association Between H3K4me3 and Transcriptional Regulation in Response to Darkness

Through detailed analysis of genes with significant changes in epigenetic marks, we found that different epigenetic marks were involved in different functions. In a previous study, we found that DHSs were mainly diminished in response to dark stress. These diminished DHSs are associated with downregulated expression of photosynthesis-associated genes, which results in loss of energy. Both DHSs and H3K4me3 have a positive relationship with gene expression. However, in this study, we found that H3K4me3’s changes in response to dark stress were different from those of DHSs. Through SOM analysis, we found that H3K4me3 was increased under dark stress. We identified 476 genes with increased H3K4me3 and upregulated transcription levels under darkness. Though GO analyses, the autophagy-associated genes were significantly enriched in these 476 genes. Based on GSEA analysis, these 476 H3K4me3-increased genes with positive relationships with expression were enriched in the gene sets of increased-regulation by KIN10, including autophagy-associated genes. KIN10 (also known as SNRK1.1), which encodes a SNF1-related protein kinase, plays an important role in linking stress, sugar and developmental signals to promote survival under darkness and sugar deprivation (Baena-Gonzalez et al., 2007). Energy deprivation leads to the activation of SnRK1-dependent signaling, which induces bZIP63 to overcome energy starvation during dark-induced leaf senescence (Liebsch and Keech, 2016). Our transcriptomic data showed that the expression of *bZIP63* was upregulated under dark stress, which also suggested that dark stress may affect SnRK1-dependent signaling. In addition, we also found that senescence-associated genes were enriched in 476 H3K4me3-increased and upregulated genes under dark stress by GO enrichment analysis. In addition, we found crosstalk for H3K4me3 between dark-induced leaf senescence and age-triggered leaf senescence. Hence, increased H3K4me3 upregulated autophagy- and senescence-associated gene expression. The functions of H3K4me3-changed genes indicated that the H3K4me3 modifications were increased but DHSs were diminished under extended darkness, although DHSs and H3K4me3 were both positive with gene expression. We investigated the DHS’s signals of the 476 genes with increased H3K4me3 and upregulation under dark stress, interestingly, these genes showed slightly diminished DHSs (Supplementary Figure 6A). We also investigated our previously identified 519 DHS-diminished and down-regulated genes under dark stress (Liu et al., 2017), there was no observable difference in the H3K4me3 signals around those genes’ regions (Supplementary Figure 6B). The diminished DHSs were associated with downregulated expression of photosynthesis-associated genes (Liu et al., 2017), whereas these increased H3K4me3 modifications were associated with upregulated expression of autophagy- and senescence-associated genes.

## Conclusion

In summary, we found that after extended dark treatment, the signals of H3K4me3 were increased, and DHS was diminished by ChIP-seq analysis, including SOM analysis. The H3K4me3 changes were associated with upregulation in the expression of autophagy- and senescence-associated genes. The H3K4me3 changes showed cross-talk between dark-induced leaf senescence and age-triggered leaf senescence. Our study revealed the expression of regulatory mechanisms related to H3K4me3 under dark stress at epigenetic levels. More epigenomic profilings are needed in the future to explore the complicated regulatory mechanisms of the plant response to dark stress.

## Data Availability

The datasets generated for this study can be found in SRA database of NCBI (accession number: PRJNA520815).

## Author Contributions

WX and ZS conceived and designed the experiments. HY, YL, KZ, and WX performed the experiments. YL, WX, and ZS analyzed the data. YL, HY, JS, and ZS contributed bioinformatics platform and analysis tools. YL, HY, WX, and ZS wrote the manuscript.

## Conflict of Interest Statement

The authors declare that the research was conducted in the absence of any commercial or financial relationships that could be construed as a potential conflict of interest.
