# Supplementary material for: Chromatin State-Based Analysis of Epigenetic H3K4me3 Marks of Arabidopsis in Response to Dark Stress
Source: Front Genet. 2019 Apr 3;10:306. doi: 10.3389/fgene.2019.00306 (PMC6456666; doi:10.3389/fgene.2019.00306)
Supplement: Supplementary file 1 [file Data_Sheet_1.doc]

**Chromatin state-based analysis of epigenetic H3K4me3 marks of *Arabidopsis* in response to dark stress**

Hengyu Yan^1^, Yue Liu^1,2†^, Kang Zhang^1,3^, James Song^4^, Wenying Xu^1^*, Zhen Su^1^*

^1^ State Key Laboratory of Plant Physiology and Biochemistry, College of Biological Sciences, China Agricultural University, Beijing 100193, China

^2^ College of Life Sciences, Qingdao University, Qingdao, Shandong 266071, China

^3^ Key Laboratory of Hebei Province for Plant Physiology and Molecular Pathology, College of Life Sciences, Hebei Agricultural University, Baoding, Hebei 071000, China, China

^4^ Henan Experimental High School, Zhengzhou, Henan 450002, China

^†^ These authors contributed equally to this work

*Authors for correspondence

Zhen Su

E-mail: [zhensu@cau.edu.cn](mailto:zhensu@cau.edu.cn)

Work Phone: +86-10-62731380

Fax: +86-10-62731380

Wenying Xu

E-mail: x_wenying@yahoo.com

Work Phone: +86-10-62731380

Fax: +86-10-62731380

**SUPPLEMENTAL FIGURES AND TABLES**

**
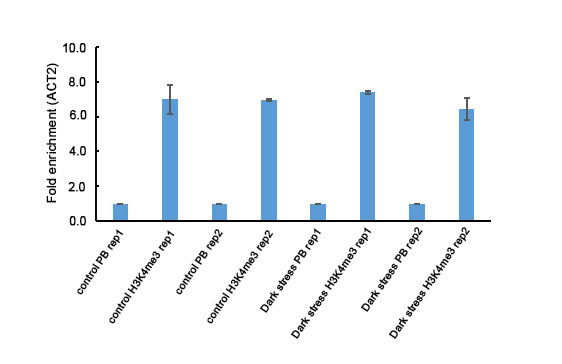
**

**Supplemental Figure 1. Relative enrichment of H3K4me3 around *ACT2* in control and dark stress sample.**

**
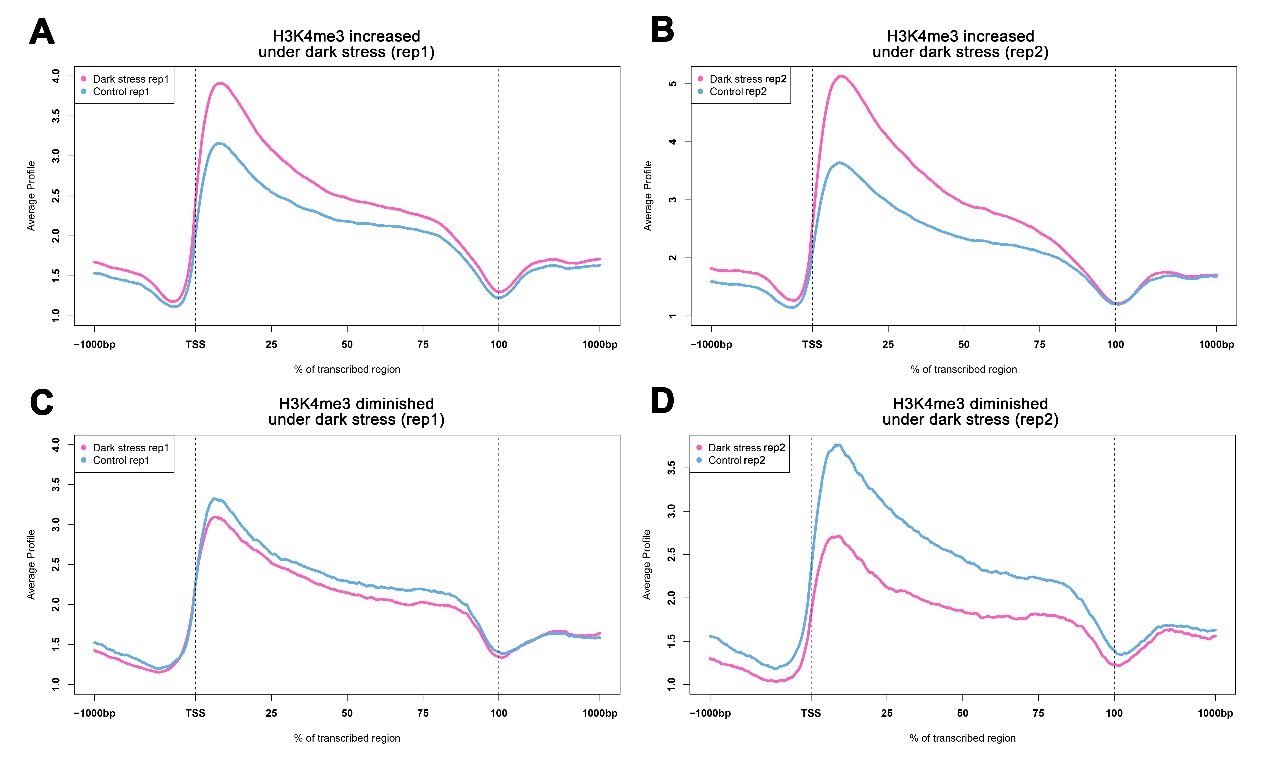
**

**Supplemental Figure 2. Average profiles of H3K4me3 signals for H3K4me3-inceased (A-B) and H3K4me3-diminished (C-D) genes.**

**(A and C)** Biological replication 1. **(B and D)** Biological replication 2.


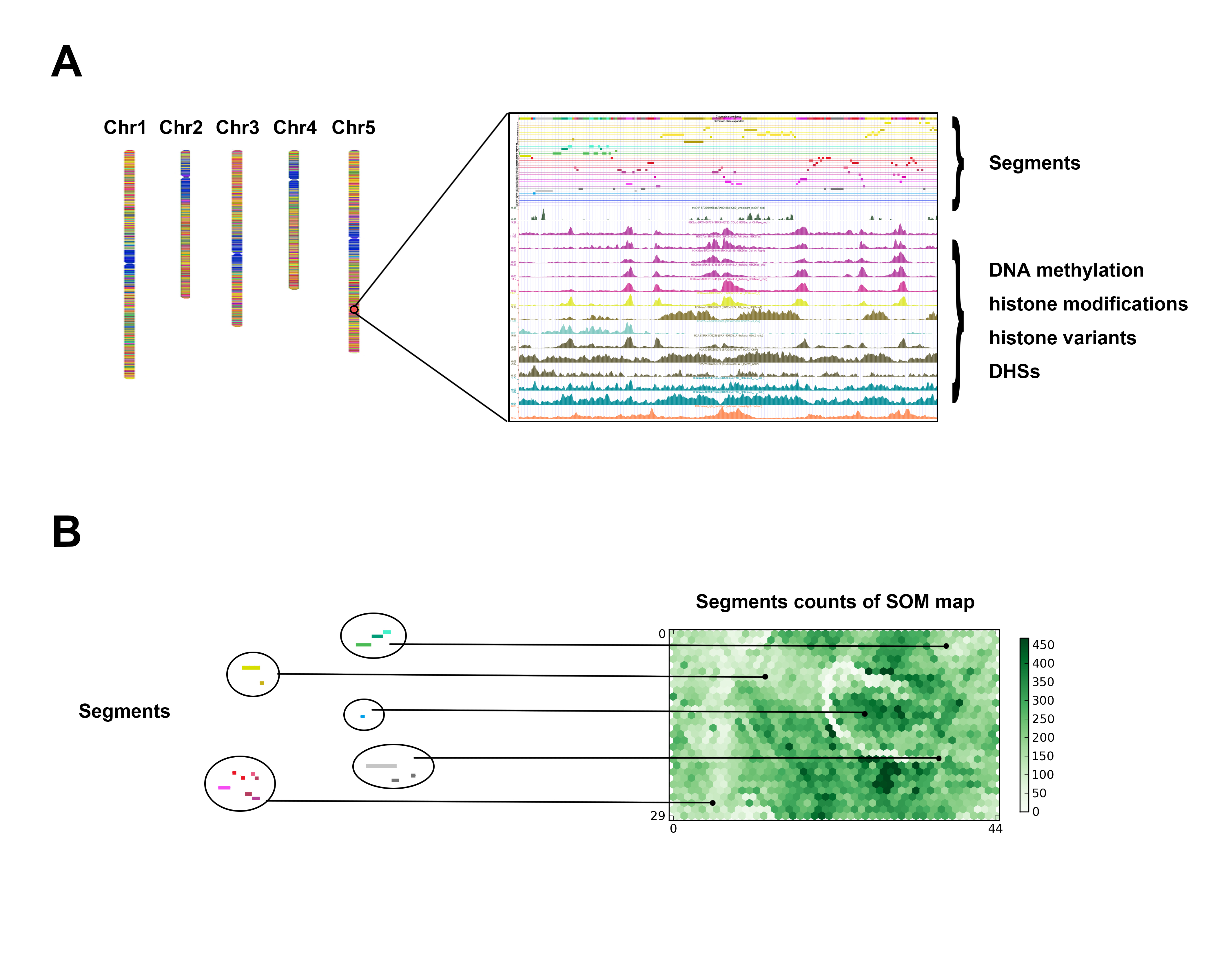


**Supplemental Figure 3.** **Chromatin states-based SOM training.**

**(A)** ChromHMM-derived genome segmentation.

**(B)** SOM training based on signal density for each segment across the genome.


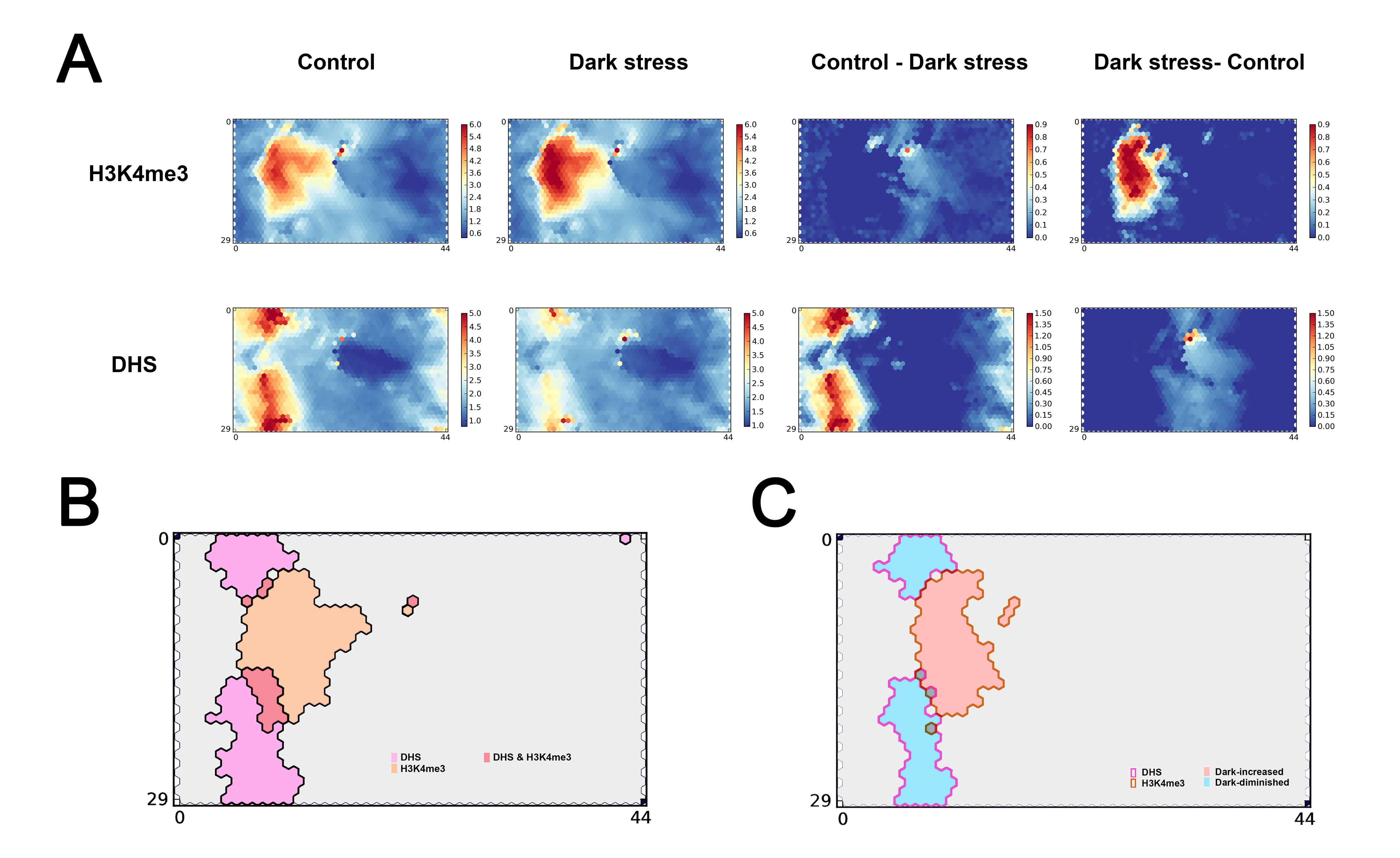


**Supplemental Figure 4. The SOM analysis for H3K4me3 and DHS signals in response to extended darkness.**

**(A)** Chromatin state-based SOM maps of H3K4me3 and DHS in response to darkness. SOM maps and differential SOM maps of H3K4me3 and DHS between dark treatment and control conditions. The ChIP-seq and DNase-seq data in different conditions are mapped to a trained SOM map in PCSD, which represents a re-organizing genome through clustering genome segments based on multiple epigenetic signals. “Control - Dark” represents the result of subtracting signals under the dark treatment from signals under the control conditions. “Dark - Control” represents the result of subtracting signals under the control conditions from signals under the dark treatment.

**(B)** SOM map-based schematic diagram representing the relationship between H3K4me3 and DHS.

**(C)** SOM map-based schematic diagram representing changes in H3K4me3 and DHS in response to dark stress.


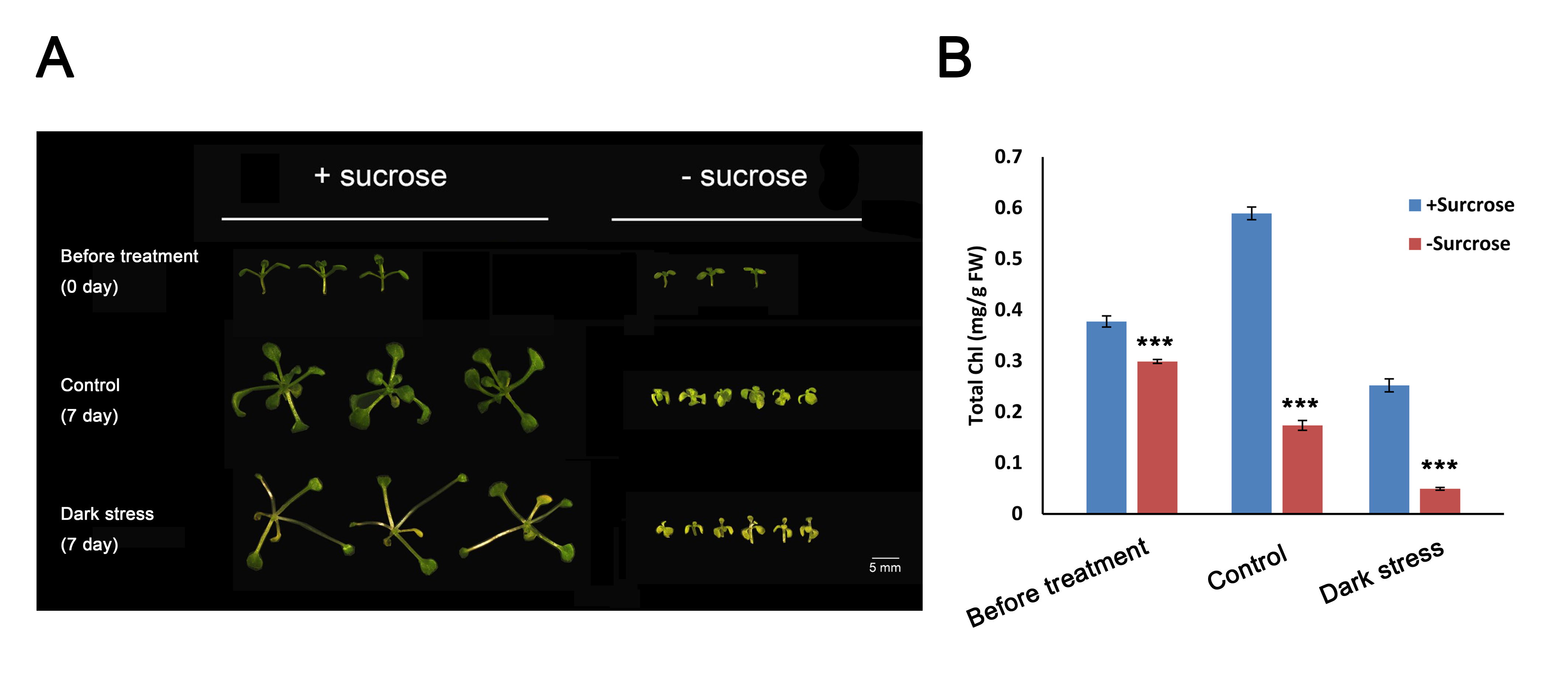


**Supplemental Figure 5.** **Sucrose effect on dark-induced leaf senescence.**

(**A**) Phenotypic photos of *Arabidopsis* leaves on culture media with different sucrose concentrations in response to dark treatment. “+Sucrose” indicates plants grown on plates with 1/2 MS; “-Sucrose” indicates plants grown on plates with no sucrose.

(**B**) Total Chl levels of *Arabidopsis* leaves on culture media with different sucrose concentrations under darkness and normal light conditions. * Indicates a significant difference between the dark stress treatment and the control under each condition. ***P <= 0.001 (Student’s t-test).


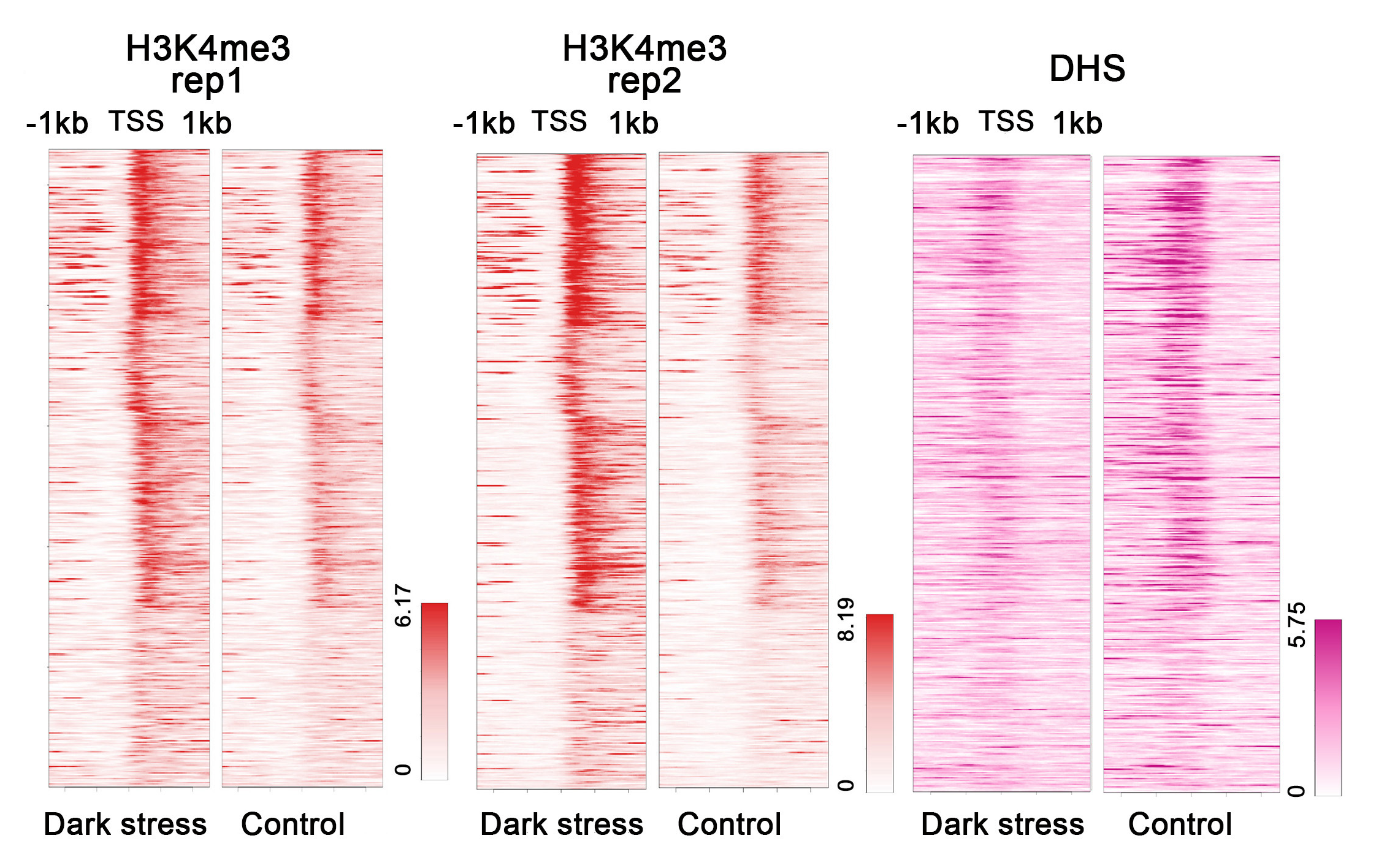


A

B


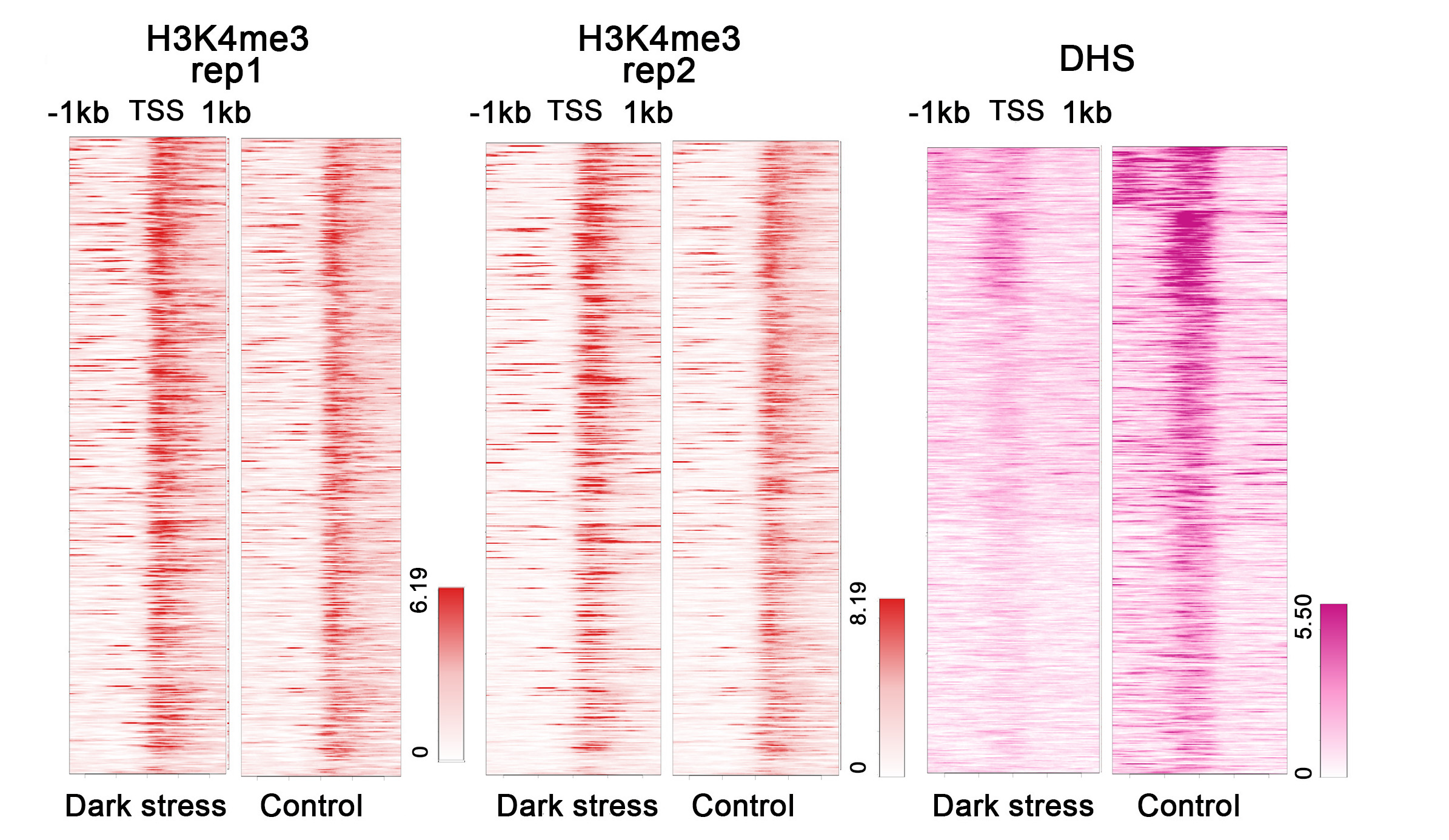


**Supplemental Figure 6. H3K4me3 and DHS signal values around TSSs of the group of 476 H3K4m3-increased and upregulated genes (A) and the group of 519 DHS-diminished and downregulated genes (B).**

The 519 genes were from our previous published articles (Liu et al., 2017). For each gene, the H3K4me3 and DHS signals are displayed along -1 kb to 1 kb regions around the TSSs.

**Supplemental Table 1.** Summary of histone modification ChIP-seq libraries.

| **Sample** | **Total reads (M)** | **Mapped Reads (M)** | **%mapping** | **Enriched regions** |
| --- | --- | --- | --- | --- |
| H3K4me3 rep1 (3-day darkness) | 57 | 54 | 96.21 | 14,827 |
| H3K4me3 rep2 (3-day darkness) | 28 | 27 | 94.5 | 13,758 |
| H3K4me3 rep1 (Control) | 42 | 41 | 97.71 | 13,921 |
| H3K4me3 rep2 (Control) | 30 | 29 | 97.57 | 13,469 |

**Supplemental Table 2.** H3K4me3-changed genes and closest differential peaks after dark treatment. (in separate Excel file Table 1.XLS)

**Supplemental Table 3. Selected enriched GO terms among H3K4me3-increased genes after dark treatment.**

| **GO term** | **Description** | **FDR** |
| --- | --- | --- |
| GO:0009738 | abscisic acid mediated signaling pathway | 3.80E-08 |
| GO:0009414 | response to water deprivation | 9.80E-08 |
| GO:0007568 | aging | 1.90E-07 |
| GO:0009753 | response to jasmonic acid stimulus | 3.00E-06 |
| GO:0009723 | response to ethylene stimulus | 2.10E-05 |
| GO:0009695 | jasmonic acid biosynthetic process | 4.10E-05 |
| GO:0009867 | jasmonic acid mediated signaling pathway | 0.00016 |
| GO:0042538 | hyperosmotic salinity response | 0.00017 |
| GO:0009744 | response to sucrose stimulus | 0.00018 |
| GO:0006612 | protein targeting to membrane | 0.00069 |
| GO:0046655 | folic acid metabolic process | 0.00079 |
| GO:0043067 | regulation of programmed cell death | 0.0016 |
| GO:0070838 | divalent metal ion transport | 0.0043 |
| GO:0006865 | amino acid transport | 0.011 |
| GO:0009646 | response to absence of light | 0.011 |
| GO:0010155 | regulation of proton transport | 0.011 |
| GO:0010150 | leaf senescence | 0.017 |
| GO:0019344 | cysteine biosynthetic process | 0.021 |
| GO:0055080 | cation homeostasis | 0.029 |

**Supplemental Table 4.** Selected enriched GO terms among H3K4me3-diminished genes after dark treatment.

| **GO term** | **Description** | **FDR** |
| --- | --- | --- |
| GO:0009739 | response to gibberellin stimulus | 1.10E-02 |
| GO:0009411 | response to UV | 1.20E-02 |
| GO:0010264 | myo-inositol hexakisphosphate biosynthetic process | 1.80E-02 |
| GO:0019761 | glucosinolate biosynthetic process | 1.80E-02 |
| GO:0010073 | meristem maintenance | 0.024 |
| GO:0007166 | cell surface receptor linked signaling pathway | 0.032 |
| GO:0006733 | oxidoreduction coenzyme metabolic process | 0.039 |
| GO:0071554 | cell wall organization or biogenesis | 0.041 |
| GO:0043086 | negative regulation of catalytic activity | 0.041 |
| GO:0006468 | protein amino acid phosphorylation | 0.05 |

**Supplemental Table 5.** Primers used for ChIP-qPCR.

| **Locus ID** | **Gene name** | **Forward Primer** | **Reverse Primer** |
| --- | --- | --- | --- |
| AT1G80840 | WRKY40 | TCCACAAGAACCCATATCGA | ACTCGCATACGAGTAACGCC |
| AT4G04620 | ATG8B | TTCTTCTCCATGATGATTCGG | CGAGTTTTTGTGGTTTGGGT |
| AT4G10570 | UBP9 | CGATTCGCTAGTAAGCTCCG | GCTCCTCTTCGAAGATGACG |
| AT5G65210 | TGA1 | TCCCTTCTGTTTATGGTCGG | TGTCGTCGTGAAAACCATGT |
| AT3G18780 | ACT2*  25S | GCGACTTGACAGAGAAGAAC  CAGTACGAATACGAACCGTG | GAAAGAGCGGAAGAAGATGAG  CAATGATAGGAAGAGCCGAC |

*: the primer sequences were adopted from Brusslan et al., 2012

**Supplemental Table 6.** Summary of RNA-seq libraries.

| **Sample** | **Total reads (M)** | **Mapped Reads (M)** | **%mapping** |
| --- | --- | --- | --- |
| RNA-seq 3-day darkness | 44 | 41 | 93.55 |
| RNA-seq Control | 36 | 34 | 93.98 |

**Supplemental Table 7.** Differentially expressed genes in response to darkness. (in separate Excel file Table 1.XLS)

**Supplemental Table 8.** Genes with a positive relationship between H3K4me3 and expression. (in separate Excel file Table 1.XLS)

**Supplemental Table 9.** Selected enriched GO terms among H3K4me3-increased and upregulated genes after dark treatment.

| **GO term** | **GO name** | **Qnum** | **B/Rnum** | **FDR** |
| --- | --- | --- | --- | --- |
| GO:0009738 | abscisic acid mediated signaling pathway | 30 | 252 | 5.10E-13 |
| GO:0007568 | aging | 20 | 145 | 1.40E-09 |
| GO:0009723 | response to ethylene stimulus | 28 | 353 | 1.50E-08 |
| GO:0010583 | response to cyclopentenone | 18 | 148 | 6.90E-08 |
| GO:0042538 | hyperosmotic salinity response | 18 | 162 | 1.80E-07 |
| GO:0009611 | response to wounding | 25 | 340 | 3.20E-07 |
| GO:0010149 | senescence | 14 | 96 | 4.30E-07 |
| GO:0009407 | toxin catabolic process | 18 | 211 | 4.20E-06 |
| GO:0046482 | para-aminobenzoic acid metabolic process | 8 | 38 | 5.10E-05 |
| GO:0009867 | jasmonic acid mediated signaling pathway | 18 | 282 | 0.00017 |
| GO:0009646 | response to absence of light | 7 | 37 | 0.00036 |
| GO:0006952 | defense response | 52 | 1653 | 0.00064 |
| GO:0006865 | amino acid transport | 16 | 266 | 0.00091 |
| GO:0009733 | response to auxin stimulus | 20 | 431 | 0.0026 |
| GO:0015706 | nitrate transport | 13 | 207 | 0.003 |
| GO:0006979 | response to oxidative stress | 24 | 582 | 0.003 |
| GO:0009744 | response to sucrose stimulus | 13 | 210 | 0.0033 |
| GO:0009863 | salicylic acid mediated signaling pathway | 17 | 349 | 0.0046 |
| GO:0010286 | heat acclimation | 8 | 84 | 0.0047 |
| GO:0009063 | cellular amino acid catabolic process | 11 | 178 | 0.0096 |
| GO:0015698 | inorganic anion transport | 13 | 266 | 0.022 |
| GO:0006986 | response to unfolded protein | 10 | 187 | 0.04 |
| GO:0010363 | regulation of plant-type hypersensitive response | 15 | 371 | 0.048 |
| GO:0006914 | autophagy | 6 | 74 | 0.049 |

**Supplemental Table 10.** Senescence-associated genes among H3K4me3-increased and upregulated genes after dark treatment. (in separate Excel file Table 1.XLS)

**Supplemental Table 11.** Autophagy-associated genes among H3K4me3-increased and upregulated genes after dark treatment.

| **Protein complexes** | **Gene accession numbers** | **Proteins** | **Expression changes under dark** | **H3K4me3 changes under dark** | **Functions and characteristics in yeast** |
| --- | --- | --- | --- | --- | --- |
| **ATG1 complex** | |  |  |  | **Initiation of autophagy** |
|  | AT1G49180 | ATG1a | up-regulated |  | Serine/threonine kinase |
|  | AT2G37840 | ATG1b | up-regulated |  | Serine/threonine kinase |
|  | AT3G53930 | ATG1c | up-regulated |  | Serine/threonine kinase |
|  | AT3G49590 | ATG13b | up-regulated |  | Phosphoprotein |
|  | AT5G06140 | ATG20 | up-regulated |  |  |
| **ATG9 complex** | |  |  |  | **Membrane recruitment to autophagosome** |
|  | AT2G31260 | ATG9 | up-regulated |  | Integral membrane protein |
|  | AT2G40810 | ATG18c | up-regulated |  | ATG9 recycling from PAS |
|  | AT5G54730 | ATG18f | up-regulated |  | ATG9 recycling from PAS |
| **PI3K complex** | |  |  |  | **Autophagosome formation** |
|  | AT3G61710 | ATG6 | up-regulated |  | VPS30 |
|  | AT4G29380 | VPS15 | up-regulated |  | Protein kinase |
| **Ubiquitin-like ATG12 and ATG5 conjugation pathway** | | | |  | **conjugation (ATG12) Conjugation of ATG12 and ATG5** |
|  | AT5G17290 | ATG5 | up-regulated |  | Target for ATG12 conjugation |
|  | AT5G45900 | ATG7 | up-regulated |  | E1 conjugating enzyme for ATG12 conjugation |
|  | AT3G13970 | ATG12b |  |  | Ubiquitin-like modifier, ATG5and ATG10interaction |
|  | AT5G50230 | ATG16 | up-regulated | increased |  |
| **Ubiquitin-like ATG8 and PE conjugation pathway** | | | |  | **Conjugation of ATG8 to phosphatidylethanolamine** |
|  | AT5G61500 | ATG3 | up-regulated |  | E2 conjugating enzyme for ATG8 conjugation |
|  | AT2G44140 | ATG4a | up-regulated |  | Cysteine protease |
|  | AT5G45900 | ATG7 | up-regulated |  | E1 conjugating enzyme for ATG8 conjugation |
|  | AT4G21980 | ATG8a | up-regulated |  | Ubiquitin-like modifier, ATG3 interaction |
|  | AT4G04620 | ATG8b | up-regulated | increased | Ubiquitin-like modifier, ATG3 interaction |
|  | AT1G62040 | ATG8c | up-regulated | increased | Ubiquitin-like modifier, ATG3 interaction |
|  | AT2G45170 | ATG8e | up-regulated |  | Ubiquitin-like modifier, ATG3 interaction |
|  | AT4G16520 | ATG8f | up-regulated |  | Ubiquitin-like modifier, ATG3 interaction |
|  | AT3G60640 | ATG8g |  |  | Ubiquitin-like modifier, ATG3 interaction |
|  | AT3G06420 | ATG8h | up-regulated | increased | Ubiquitin-like modifier, ATG3 interaction |
|  | AT3G15580 | ATG8i | up-regulated |  | Ubiquitin-like modifier, ATG3 interaction |
| SNARE |  |  |  |  | **Fusion of autophagososme with the vacuole** |
|  | AT1G26670 | VTI12 | up-regulated |  |  |
| Others involved in autophagy | | |  |  |  |
|  | AT4G23450 | AIRP1 | up-regulated | increased |  |
|  | AT5G02020 | SIS | up-regulated | increased |  |
|  | AT5G49690 | | up-regulated | increased |  |

**Supplemental Table 12.** Selected enriched gene sets from literature among H3K4me3-increased and upregulated genes after dark treatment.

| Description | NO. Genes in Overlap | FDR |
| --- | --- | --- |
| Induced by KIN10 markedly overlaps with that induced by starvation conditions and is antagonized by increased sugar availability. (Table S4 PubmedID:17671505) | 73 | 5.34E-55 |
| Increased-regulation by KIN10. (Table S3 PubmedID:17671505) | 88 | 2.34E-54 |

**Supplemental Table 13.** KIN10-induced genes that were upregulated and H3K4me3-increased during dark-induced leaf senescence. (in separate Excel file Table 1.XLS)

**Supplemental Table 14.** Selected enriched GO terms among H3K4me3-diminished and downregulated genes after dark treatment.

| **GO term** | **GO name** | **Qnum** | **B/Rnum** | **FDR** |
| --- | --- | --- | --- | --- |
| GO:0019252 | starch biosynthetic process | 10 | 191 | 0.0012 |
| GO:0016556 | mRNA modification | 7 | 101 | 0.0042 |
| GO:0046148 | pigment biosynthetic process | 11 | 282 | 0.0044 |
| GO:0009411 | response to UV | 10 | 247 | 0.0065 |
| GO:0006733 | oxidoreduction coenzyme metabolic process | 10 | 267 | 0.011 |
| GO:0009069 | serine family amino acid metabolic process | 10 | 271 | 0.011 |
| GO:0006725 | cellular aromatic compound metabolic process | 21 | 1022 | 0.016 |
| GO:0010033 | response to organic substance | 41 | 2754 | 0.021 |
| GO:0006468 | protein amino acid phosphorylation | 22 | 1134 | 0.021 |
| GO:0010264 | myo-inositol hexakisphosphate biosynthetic process | 5 | 65 | 0.021 |
| GO:0000023 | maltose metabolic process | 7 | 152 | 0.024 |
| GO:0043086 | negative regulation of catalytic activity | 6 | 112 | 0.027 |
| GO:0007166 | cell surface receptor linked signaling pathway | 7 | 160 | 0.029 |
| GO:0010374 | stomatal complex development | 8 | 217 | 0.032 |
| GO:0009310 | amine catabolic process | 8 | 214 | 0.032 |
| GO:0010075 | regulation of meristem growth | 7 | 171 | 0.036 |
| GO:0071555 | cell wall organization | 14 | 613 | 0.041 |

**Supplemental Table 15.** H3K4m3-increased genes under dark-induced and age-triggered senescence. (in separate Excel file Table 1.XLS)

**Supplemental Table 16.** H3K4m3-diminished genes under dark-induced and age-triggered senescence. (in separate Excel file Table 1.XLS)
